# Ocular higher-order aberrations and axial eye growth in young Hong Kong children

**DOI:** 10.1038/s41598-018-24906-x

**Published:** 2018-04-30

**Authors:** Jason K. Lau, Stephen J. Vincent, Michael J. Collins, Sin-Wan Cheung, Pauline Cho

**Affiliations:** 1 Centre for Myopia Research, School of Optometry, The Hong Kong Polytechnic University, Kowloon, Hong Kong SAR China; 2 Contact Lens and Visual Optics Laboratory, School of Optometry and Visual Science, Queensland University of Technology, Brisbane, Queensland Australia

## Abstract

This retrospective longitudinal analysis aimed to investigate the association between ocular higher-order aberrations (HOAs) and axial eye growth in Hong Kong children. Measures of axial length and ocular HOAs under cycloplegia were obtained annually over a two-year period from 137 subjects aged 8.8 ± 1.4 years with mean spherical equivalent refraction of −2.04 ± 2.38 D. A significant negative association was observed between the RMS of total HOAs and axial eye growth (*P* = 0.03), after adjusting for other significant predictors of axial length including age, sex and refractive error. Similar negative associations with axial elongation were found for the RMS of spherical aberrations (${{\rm{Z}}}_{4}^{0}$ and ${{\rm{Z}}}_{6}^{0}$ combined) (*P* = 0.037). Another linear mixed model also showed that greater levels of vertical trefoil $({{\rm{Z}}}_{3}^{-3})$, primary spherical aberration $({{\rm{Z}}}_{4}^{0})$ and negative oblique trefoil $({{\rm{Z}}}_{3}^{3})$ were associated with slower axial elongation and longer axial length (all *P* < 0.05). These findings support the potential role of HOAs, image quality and a vision-dependent mechanism in childhood eye growth.

## Introduction

Myopia is a global health concern and a leading cause of visual impairment^1^ with reports of increasing prevalence and frequent rapid progression, particularly in East Asian countries^2^. The increase in axial length, associated with myopia development and progression^3^, results in an elongation of the posterior eye^4^ and consequently poses a higher risk for the development of pathological changes in the retina and choroid in later life^5^.

Visual experience is thought to play a substantial role in the regulation of axial eye growth since it can be predictably altered in young animals reared with lens induced defocus, confined environments with restricted viewing distances, or form deprivation^6–8^. Similarly, humans also display a transient change in axial length and choroidal thickness (although smaller in magnitude) following short-term exposure to defocus^9–11^. Since the eyes of a number of species can detect and rapidly respond to imposed defocus in a bi-directional manner to minimize blur, this suggests that a vision-dependent mechanism may underlie the emmetropisation process and refractive error development during childhood.

While defocus and astigmatism (lower-order aberrations) can be corrected with conventional spectacles or contact lenses, other optical imperfections described as higher-order aberrations (HOAs) cannot be corrected using traditional optical corrections and have the potential to alter retinal image quality^12,13^ which may provide cues to the retina^14^, and possibly lead to the development of myopia^15^. Numerous studies have investigated the relationships between HOAs and age^16–21^, axial length^22,23^, refractive error^24^, accommodation^13,25^, and ethnicity^24,26,27^. While 3rd and 4th order aberrations increase with age in children^16^ and adults^20,28,29^, the relationship between HOAs and refractive error remains equivocal (summarised by Little *et al*.^23^). Importantly, the vast majority of these studies are cross-sectional in nature and do not provide a clear understanding of the association between HOAs or image quality and axial eye growth, as aberrations may vary substantially among different individuals^17^.

To date, only three studies have examined the change in HOAs throughout childhood. Zhang *et al*.^30^ found a weak but statistically significant positive correlation between HOAs and myopia progression in their cohort of myopes (mean age: 12.1 years). They further compared individual Zernike coefficients in fast and slow progressors and showed that more coma and trefoil were associated with slower myopia progression. On the contrary, Philip *et al*.^31^ reported no significant association between HOAs and myopia development in primary emmetropes over a 5-year period (mean age: 12.6 years). However, in subjects who demonstrated myopia progression, positive spherical aberration decreased over time but the opposite trend was found in subjects who remained emmetropic throughout the observation period. Recently, in a younger cohort of myopic Japanese (mean age: 9.2 years), Hiraoka *et al*.^32^ observed a significant negative association between both total corneal and ocular HOAs and axial eye growth. They also demonstrated a negative association between positive spherical aberration and axial elongation. However, these longitudinal studies often failed to adjust for other known potential confounders of HOAs such as age, axial length and refractive error, and consequently, the relationship between HOAs and axial eye growth in children remains uncertain.

Thus, the primary aim of this retrospective longitudinal study was to further the current understanding of the association between ocular HOAs and axial length and to examine the relationship between HOAs and axial eye growth over a two-year period in young Hong Kong children.

## Results

For all analyses, only subjects with cycloplegic refraction, wavefront aberrations and axial length data over two years of follow-up were included. The HOAs of one subject was not analysed due to poor COAS image quality and was excluded. This yielded a final sample size of 137 subjects. Participants with pupil diameters of less than 6 mm were excluded from the analysis at these specific visits, since extrapolation of the wavefront data to a larger pupil size is associated with some errors^33^ and the linear mixed model (LMM) statistical approach accounts for individual missing data^34^. Root-mean-square (RMS) errors (square root of the sum of the squares of corresponding Zernike coefficients) indicate the variance of an aberrated wavefront in comparison to an ideal wavefront (similar to the standard deviation) and is a commonly used measure of optical quality. In the LMMs, we employed the RMS of total higher-order aberrations (3rd to 6th orders inclusive, HO RMS) (Model 1), RMS of spherical-like aberrations (SA) (${{\rm{Z}}}_{4}^{0}$ and ${{\rm{Z}}}_{6}^{0}$ combined, SA RMS), RMS of coma-like aberrations (${{\rm{Z}}}_{3}^{-1}$, ${{\rm{Z}}}_{3}^{1}$, ${{\rm{Z}}}_{5}^{-1}$ and ${{\rm{Z}}}_{5}^{-1}$ combined, coma RMS) (Model 2), and individual Zernike coefficients (Model 3) to identify specific RMS or aberration terms in association with axial length and its progression. Table 1 illustrates the baseline demographics and HOAs of the population.

**Table 1:** Baseline demographics, individual Zernike coefficients and RMS of higher-order aberrations of the pooled population.

	N	Mean ± SD	Median	Range
Demographics				
Age, y	137	8.8 ± 1.4	8.6	6.1–12.6
SER, D	137	−2.04 ± 2.38	−2.00	−8.63−+2.50
Axial length, mm	137	24.09 ± 1.24	24.10	21.35–27.06
Zernike coefficient, µm				
${{\rm{Z}}}_{3}^{-3}$	128	0.057 ± 0.120	0.054	−0.229–0.372
${{\rm{Z}}}_{3}^{-1}$	128	0.083 ± 0.175	0.064	−0.380–0.580
${{\rm{Z}}}_{3}^{1}$	128	0.004 ± 0.091	0.008	−0.226–0.225
${{\rm{Z}}}_{3}^{3}$	128	−0.019 ± 0.098	−0.029	−0.324–0.261
${{\rm{Z}}}_{4}^{-4}$	128	0.027 ± 0.033	0.027	−0.099–0.117
${{\rm{Z}}}_{4}^{-2}$	128	−0.023 ± 0.031	−0.021	−0.181–0.072
${{\rm{Z}}}_{4}^{0}$	128	0.076 ± 0.108	0.069	−0.174–0.349
${{\rm{Z}}}_{4}^{2}$	128	0.014 ± 0.061	0.020	−0.148–0.338
${{\rm{Z}}}_{4}^{4}$	128	0.026 ± 0.051	0.021	−0.093–0.268
${{\rm{Z}}}_{5}^{-5}$	128	−0.012 ± 0.021	−0.011	−0.091–0.046
${{\rm{Z}}}_{5}^{-3}$	128	0.001 ± 0.025	0.002	−0.153–0.080
${{\rm{Z}}}_{5}^{-1}$	128	0.015 ± 0.030	0.013	−0.091–0.098
${{\rm{Z}}}_{5}^{1}$	128	0.002 ± 0.016	0.002	−0.047–0.049
${{\rm{Z}}}_{5}^{3}$	128	0.005 ± 0.014	0.005	−0.037–0.043
${{\rm{Z}}}_{5}^{5}$	128	0.008 ± 0.020	0.008	−0.085–0.060
${{\rm{Z}}}_{6}^{-6}$	128	0.000 ± 0.013	0.000	−0.042–0.047
${{\rm{Z}}}_{6}^{-4}$	128	−0.005 ± 0.010	−0.003	−0.060–0.017
${{\rm{Z}}}_{6}^{-2}$	128	0.000 ± 0.008	0.001	−0.021–0.028
${{\rm{Z}}}_{6}^{0}$	128	−0.023 ± 0.018	−0.024	−0.076–0.042
${{\rm{Z}}}_{6}^{2}$	128	0.004 ± 0.014	0.002	−0.046–0.088
${{\rm{Z}}}_{6}^{4}$	128	−0.006 ± 0.014	−0.006	−0.042–0.069
${{\rm{Z}}}_{6}^{6}$	128	0.002 ± 0.018	0.000	−0.046–0.127
RMS, µm				
HO RMS	128	0.320 ± 0.105	0.292	0.133–0.674
SA RMS	128	0.112 ± 0.076	0.091	0.015–0.352
Coma RMS	128	0.185 ± 0.112	0.159	0.023–0.583

SER: spherical equivalent refraction; HO RMS: RMS of total higher-order aberrations (3rd to 6th orders inclusive); SA RMS: RMS of spherical-like aberrations (${{\rm{Z}}}_{4}^{0}$ and ${{\rm{Z}}}_{6}^{0}$ combined); coma RMS: RMS of coma-like aberrations (${{\rm{Z}}}_{3}^{-1}$, ${{\rm{Z}}}_{3}^{1}$, ${{\rm{Z}}}_{5}^{-1}$ and ${{\rm{Z}}}_{5}^{-1}$ combined).

### Model 1: HO RMS and axial length

The LMM revealed significant effects of age, sex and SER on axial length (Table 2). As expected, axial length, increased with age (*P* < 0.001), was shorter in girls compared to boys (0.67 mm shorter, *P* < 0.001) and was greater in subjects with higher myopia (0.32 mm greater per 1 D myopia, *P* < 0.001). Greater levels of HO RMS were associated with a longer axial length and a slower annual axial growth rate (0.2 mm longer and 0.1 mm/y decrease per 0.1 µm HO RMS, respectively, *P* < 0.05).

**Table 2:** Statistically significant fixed effects and parameter estimates of the influences on change in axial length.

Parameter	All subjects (n = 137)		Myopes only (n = 113)	
	Parameter estimates	*P* value	Parameter estimates	*P* value
Model 1 – HO RMS				
Intercept	20.23	**<0.001**	19.96	**<0.001**
ln(age)	1.68	**<0.001**	1.80	**<0.001**
Sex*	−0.67	**<0.001**	−0.60	**<0.001**
SER	−0.32	**<0.001**	−0.31	**<0.001**
HO RMS^†^	0.20	**0.046**	0.22	0.071
Time by HO RMS^†^	−0.10	**0.030**	−0.11	**0.048**
Model 2 – SA RMS and coma RMS				
Intercept	20.51	**<0.001**	20.21	**<0.001**
ln(age)	1.55	**<0.001**	1.68	**<0.001**
Sex*	−0.67	**<0.001**	−0.60	**<0.001**
SER	−0.31	**<0.001**	−0.31	**<0.001**
Time by SA RMS^†^	−0.13	**0.037**	−0.16	**0.037**
Model 3 – individual Zernike terms				
Intercept	20.29	**<0.001**	20.09	**<0.001**
ln(age)	1.64	**<0.001**	1.75	**<0.001**
Sex*	−0.68	**<0.001**	−0.60	**<0.001**
SER	−0.31	**<0.001**	−0.30	**<0.001**
Zernike terms^†^				
${{\rm{Z}}}_{3}^{-3}$	0.25	**0.011**	0.22	0.085
${{\rm{Z}}}_{3}^{3}$	−0.28	**0.041**	−0.23	0.170
${{\rm{Z}}}_{4}^{0}$	0.26	**0.032**	0.33	**0.031**
Time by Zernike terms^†^				
Time by ${{\rm{Z}}}_{3}^{-3}$	−0.11	**0.012**	−0.10	0.064
Time by ${{\rm{Z}}}_{3}^{3}$	0.13	**0.033**	0.11	0.122
Time by ${{\rm{Z}}}_{4}^{0}$	−0.11	**0.032**	−0.14	**0.042**

Other parameters and interactions with time did not show statistically significant effects (all *P* > 0.05) in the LMMs.

SER: spherical equivalent refraction; HO RMS: RMS of total higher-order aberrations (3rd to 6th orders inclusive); SA RMS: RMS of spherical-like aberrations (${{\rm{Z}}}_{4}^{0}$ and ${{\rm{Z}}}_{6}^{0}$ combined); coma RMS: RMS of coma-like aberrations (${{\rm{Z}}}_{3}^{-1}$, ${{\rm{Z}}}_{3}^{1}$, ${{\rm{Z}}}_{5}^{-1}$ and ${{\rm{Z}}}_{5}^{-1}$ combined).

*Parameter estimate for girls. ^†^Per 0.1 µm.

### Model 2: SA RMS & coma RMS and axial length

Further analyses were performed to investigate the association between SA RMS and coma RMS on axial length (Table 2). Higher levels of SA RMS were associated with slower axial elongation (0.13 mm/y slower per 0.1 µm SA RMS, *P* = 0.037). However, there was no association between coma RMS and axial length nor axial eye growth (*P* > 0.05).

### Model 3: Individual Zernike coefficients and axial length

The LMM analyses evaluating the effects of individual Zernike terms on axial length revealed that more positive vertical trefoil $({{\rm{Z}}}_{3}^{-3})$ and primary SA $({{\rm{Z}}}_{4}^{0})$ and negative oblique trefoil $({{\rm{Z}}}_{3}^{3})$ were associated with a longer axial length (${{\rm{Z}}}_{3}^{-3}$: β = 0.25, *P* = 0.011; ${{\rm{Z}}}_{4}^{0}$: β = 0.26, *P* = 0.032; ${{\rm{Z}}}_{3}^{3}$: β = −0.28, *P* = 0.041) (Table 2). However, greater levels of positive ${{\rm{Z}}}_{3}^{-3}$ and ${{\rm{Z}}}_{4}^{0}$ were associated with a decreased rate of axial elongation (both ${{\rm{Z}}}_{3}^{-3}$ and ${{\rm{Z}}}_{4}^{0}$: β = −0.11 mm/y slower per 0.1 µm, *P* < 0.05), while ${{\rm{Z}}}_{3}^{3}$ displayed a statistically significant positive association with axial eye growth (${{\rm{Z}}}_{3}^{3}$: β = 0.13 mm/y faster per 0.1 µm, *P* = 0.033).

**Figure 1:**
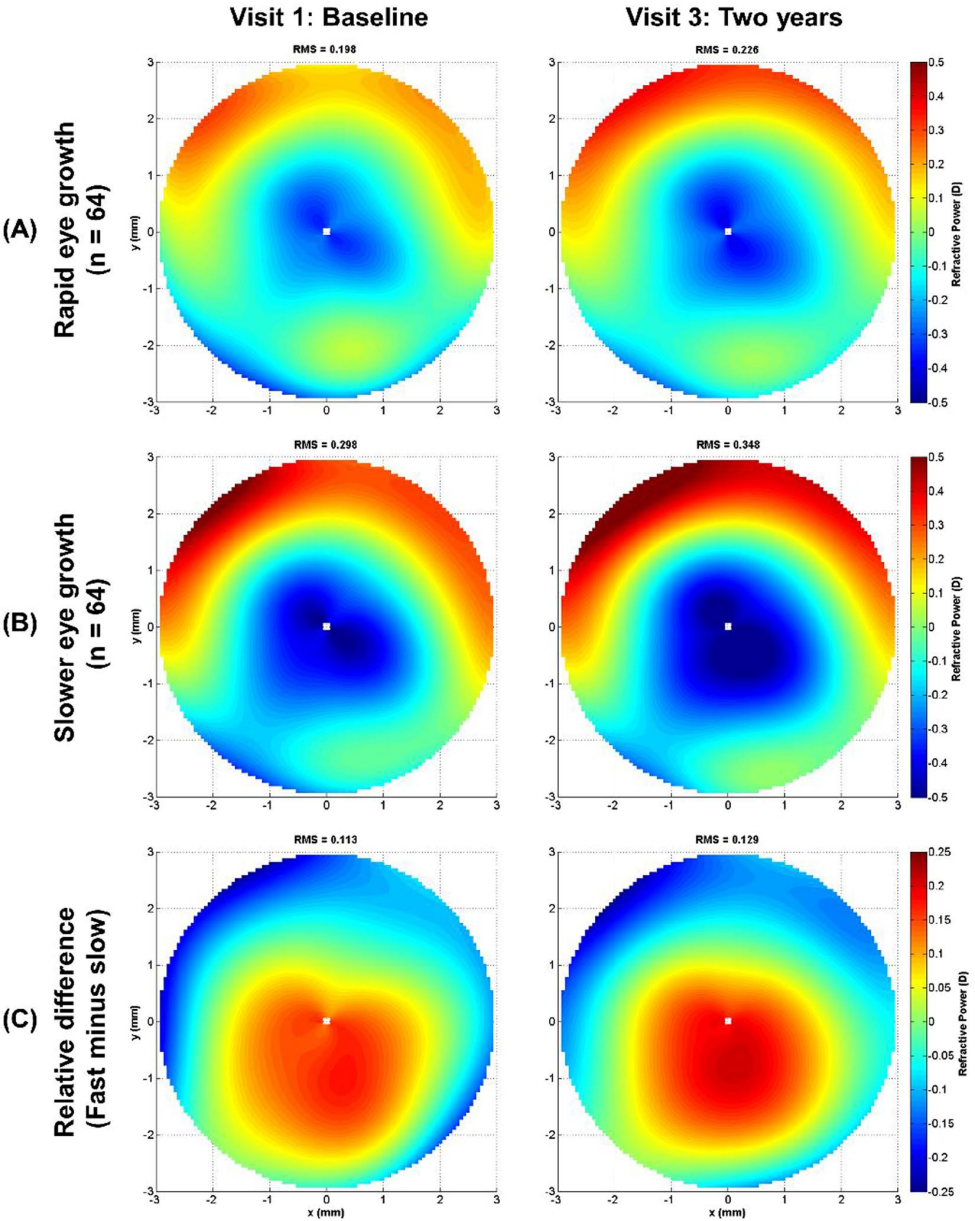
Mean refractive power maps generated from the ocular higher-order aberrations (3rd to 6th order inclusive, for a 6 mm pupil diameter) at the baseline and two-year follow up visits for subjects who exhibited (**A**) rapid eye growth (mean ± SD: 0.91 ± 0.23 mm) and (**B**) slower eye growth (mean ± SD: 0.40 ± 0.15 mm) based on a median split of the axial elongation over two years (n = 64 in each group). (**C**) Difference maps (rapid minus slower eye growth groups) highlight the relative difference in the higher order aberration profile between the two groups. Note: the refractive power scale in (**C**) differs to (**A**) and (**B**).

**Figure 2:**
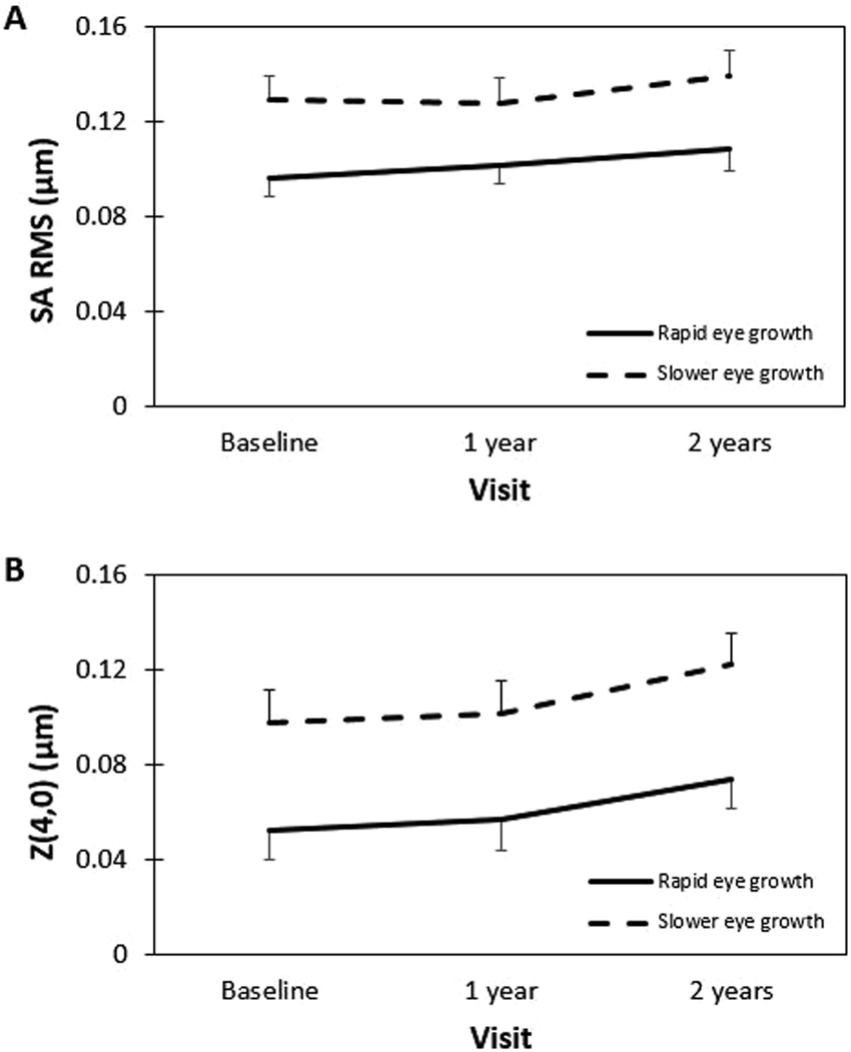
The change in unadjusted (**A**) SA RMS and (**B**) primary SA $({{\rm{Z}}}_{4}^{0})$ values over a 6 mm pupil diameter for subjects who exhibited rapid eye growth (solid, mean ± SD: 0.91 ± 0.23 mm) and slower eye growth (dashed, mean ± SD: 0.40 ± 0.15 mm) based on a median split of the axial elongation over two years (n = 64 in each group). Error bars represent the standard error. SA RMS: RMS of spherical-like aberrations (${{\rm{Z}}}_{4}^{0}$ and ${{\rm{Z}}}_{6}^{0}$ combined); SA: spherical aberration.

Given the statistically significant associations identified in the three LMMs between various HOA terms and axial elongation, refractive power maps were generated. The maps allowed visualization of differences in the ocular HOAs over a 6-mm pupil diameter (as a dioptric power map) for subjects who exhibited rapid eye growth (n = 64, mean ± SD: 0.45 ± 0.12 mm/y) and subjects who exhibited slower eye growth (n = 64, mean ± SD: 0.20 ± 0.07 mm/y) based on a median split of the data (median axial elongation of 0.625 mm over two years). Figure 1 displays the mean refractive power maps at the baseline and final study visits for the rapid and slower eye growth cohorts and the relative difference based on the median axial elongation split. On average, subjects with slower eye growth throughout the study displayed more positive SA indicated by a larger and more rapid shift towards positive powers (warmer colours) at the edge of the pupil. This is also demonstrated in Fig. 2, which displays the raw total ocular SA RMS and primary SA data (without correction for age, sex or SER) for the rapid and slower eye growth cohorts over the two-year follow-up period. It indicated that more positive SA at baseline and subsequent visits were shown in subjects exhibiting slower axial eye growth throughout the study.

## Discussion

This study, which examined the longitudinal change in axial length in young Hong Kong children, demonstrates a significant relationship between ocular HOAs and axial eye growth (when controlled for potential confounding factors), indicating that greater levels of inherent total HO RMS displayed slower axial elongation.

Only three longitudinal studies have examined the relationship between habitual HOAs and axial eye growth^30–32^. Zhang *et al*.^30^ reported that myopic children with rapid myopia progression (≥0.50 D per year) displayed significantly higher levels of HO RMS (about 0.05 µm greater) at their most recent visit, compared to subjects with slower myopia progression, which appears to be due to a difference in 3rd order aberrations between fast and slow progression groups. Their study relied on cycloplegic refraction to assess myopia progression rather than ocular biometry, and included data from both eyes which artificially inflates any statistical associations due to the high correlation between the fellow eyes and reduces probability (*P*) values^35^. In addition, measurements taken by the Zywave aberrometer (Bausch & Lomb, Rochester, NY, USA) might not provide repeatable measurements for HOAs^36,37^. Conversely, Philip *et al*.^31^ observed no significant association between HOAs at baseline and myopia development or progression in a cohort of young emmetropes. However, their subjects, who were older, showed less axial elongation (mean annual progression: 0.05 mm/y) compared to that of our subjects (mean annual progression: 0.33 mm/y), which might not justify the association between HOAs and axial elongation. Hiraoka *et al*.^32^ recently showed that higher levels of corneal HOAs were associated with less myopia progression and axial elongation. However, in these studies, HOAs were only either measured at a single visit or averaged over several visits, which might not reflect the true temporal relationship between axial length and HOAs or account for high inter-subject variability^17^. In addition, other confounding variables such as age and axial length (degree of myopia) were typically not considered in their analyses. Our study aimed to provide a better understanding of the association between HOAs and axial elongation during childhood by including measurements of both variables repeatedly over a two-year period and controlling for possible confounders in the analyses.

The mean ± SD baseline HO RMS of our population was 0.320 ± 0.105 µm (Table 1) for a 6-mm pupil, which was consistent with previous studies of myopic children of similar age (mean HO RMS: 0.304–0.462 µm; mean age: 6.7–9.2 years)^38,39^. Our findings support the hypothesis that increased levels of HOAs may influence axial eye growth by altering retinal image quality and providing a directional cue that slows axial eye growth (i.e. eye growth was 0.1 mm/y slower per 0.1 µm increase in HO RMS, *P* = 0.03). Significant associations with axial elongation were also observed in SA (SA RMS and ${{\rm{Z}}}_{4}^{0}$: both *P* < 0.05), which were in agreement with Hiraoka *et al*.^32^ who observed that, more positive SA was associated with less eye growth. Our results were also similar to the emmetropes in Philip *et al*.’s^31^ longitudinal study in which more positive SA was associated with less myopic shift. Our results were consistent with theoretical models that suggest greater levels of negative SA would produce relative peripheral hyperopic defocus and provide an optical cue for myopia progression^40^.

Hiraoka *et al*.^41^ found significant increases in corneal and total ocular HOAs after orthokeratology, a corneal reshaping intervention for controlling myopia. They found that increased levels of HO RMS, SA RMS, and coma RMS were significantly correlated with slower axial elongation. Similarly, Zhong *et al*.^42^ showed that increased levels of positive corneal peripheral power were associated with slower axial elongation in orthokeratology wear. Cheng *et al*.^43^ also found that children wearing soft contact lenses with increased positive SA displayed slower axial eye growth over a 12-month period, similar to our findings that higher levels of habitual positive SA were associated with slower axial elongation (Figs 1 and 2, Table 2).

The subjects in our study underwent cycloplegia in order to control for accommodation^44^ and ensure a large pupil diameter during COAS measurements. However, larger pupil size significantly increases the magnitude of HOAs^45,46^. The pupil size of 6 mm chosen for analysis is consistent with pupil diameters under low luminance^47^. By analysing the wavefronts over a common fixed diameter, we have eliminated this variable from the modeling. However, natural pupil variations will influence the levels of HOAs experienced by children in normal viewing conditions. Since HOAs, including SA, may change with accommodation and influence accommodation demand^48^ and accuracy^49^, further studies investigating the changes in aberrations during accommodation without cycloplegia may provide more insights into the influence of HOAs and retinal image quality on axial eye growth under habitual viewing conditions.

Trefoils (${{\rm{Z}}}_{3}^{-3}$ and ${{\rm{Z}}}_{3}^{3}$) were found to be significantly associated with axial length and axial elongation. Carkeet *et al*.^24^ found that myopes exhibit less ${{\rm{Z}}}_{3}^{-3}$ compared to emmetropes and Martinez *et al*.^50^ reported less ${{\rm{Z}}}_{3}^{-3}$ in emmetropes compared to hyperopes in contrast with our finding of a significant positive correlation between the magnitude of ${{\rm{Z}}}_{3}^{-3}$ and axial length after controlling for other variables (Table 2 [model 3]). Increased levels of ${{\rm{Z}}}_{3}^{-3}$ were also associated with slower axial eye growth (0.11 mm/y slower per 0.1 µm, *P* = 0.012, Table 2 [model 3]). Philip *et al*.^31^ monitored a cohort of emmetropes for 5 years and found that higher levels of coma RMS were observed in subjects without a myopic shift. They suggested that the changes in comatic aberrations could be due to tilts of ocular components which resulted in changes in the shape of ocular surfaces. Hiraoka *et al*.^41^ also identified an asymmetric corneal shape, in terms of corneal multifocality as one ocular parameter, was associated with slower axial elongation during orthokeratology treatment. The source and contribution of an asymmetric optical profile (e.g. trefoil) on axial eye growth through a potential local mechanism^51^ requires further investigation using instruments that can measure both corneal and internal aberrations simultaneously to identify the origins of particular aberration terms.

As expected, age was positively associated with axial length as shown in previous studies^52,53^. The LMM analysis used for predicting the change in axial length with age in our subjects was similar to that in previous ocular component modeling, showing faster axial growth in younger subjects^54^. However, in comparison to previous modelling of axial length in young emmetropes (parameter estimate: axial length = 20.19 + 1.26*ln(age) before age 10.5 years), our study showed a smaller initial axial length and a much greater axial growth rate (parameter estimate: axial length = 17.39 + 3.11*ln(age)) indicating more rapid eye growth in our subjects (80% had myopia). The change in axial length in our population was the same among boys and girls (*P* > 0.05) in contrast to a previous study that reported faster axial growth in young Singaporean females^55^. Such discrepancies may be due to ethnicity^56,57^, differences in light exposure^58^, or other genetic^59,60^ and environmental factors^58,61^ not controlled or quantified in this study. In our study, a 1 D increase in myopia was associated with a 0.32 mm increase in axial length, in agreement with other published data^62^. The axial eye growth of subjects with higher myopia was not significantly faster than those with lower myopia (*P* > 0.05). However, since our analysis was based on the pooled cohort with 80% of myopes, we did not analyse the ocular characteristics between different refractive groups.

Our findings indicate that HOAs are associated with axial elongation during childhood and may provide a visual cue that guides eye growth^14,63^. Various myopia control interventions including atropine^64,65^, multifocal soft contact lenses^66^ and ortho-k^67–69^ have been proven to be effective in controlling axial length progression in young myopic children, despite the unknown underlying optical or physiological mechanisms. However, it has been suggested that these interventions reduce accommodation amplitude^70^ or accommodative lag^71^, which would reduce the magnitude of negative spherical aberration. A positive shift in SA has also been shown in children receiving 1% atropine due to reduced accommodation and pupil dilation^72^ and ortho-k treatment due to corneal reshaping^41^ (central flattening and mid-peripheral steepening).

Most previous studies were cross-sectional and a very limited number of studies investigated the influence of HOA on axial eye growth in children. Since aberrations vary vary substantially among individuals^17^, a longitudinal study with a repeated measures design should provide a better understanding of the relationship between HOA on axial elongation. Other covariates such as age and gender, which are associated with axial length and its elongation, were also controlled in the statistical analyses.

One limitation of our study is the relatively small sample size and short follow-up period (two years). Although the current study pooled subjects from several clinical trials, the final sample was not large enough to investigate the effect of HOAs on axial eye growth in different refractive error groups, since the majority of subjects were myopic. Subjects with hyperopia, myopia and astigmatism may behave differently and our study can serve as a general observation of the effects of HOAs on axial eye growth. In addition, as the increase in axial length is a nonlinear function of age^73^, it would be of interest if further studies could investigate if there is a critical period of association between axial eye growth and ocular HOAs.

Our study addressed the relationship between higher order aberrations and myopia progression. The results (Figs 1 and 2, Table 2) showed that less axial eye growth was observed in subjects with more positive spherical aberrations at the initial baseline and subsequent visits. However, our study could not answer the question of whether higher order aberrations result in the onset of myopia, which can be only answered by measuring aberrations prior to the development of myopia. Therefore, further longitudinal studies are required to confirm the influence of higher order aberrations on the development of myopia.

In the current study, ocular HOAs were measured under cycloplegia with no optical correction in place, however, changes in HOAs are induced by habitual correction with spectacle lenses or contact lenses and during accommodation^48^. Measures of HOAs under habitual conditions (i.e. during spectacle or contact lens wear without cycloplegia and during accommodation) may provide further insights into the association between the optics of the eye and axial elongation. Furthermore, while statistically significant associations were observed between various HOAs terms and axial eye growth after controlling for confounding variables, this association does not necessarily infer a causal relationship between HOAs and axial eye growth. Future prospective studies on myopia control interventions which induce changes in HOAs, for example multifocal contact lenses or ortho-k treatment, may improve the current understanding of the relationship between HOAs and axial eye growth.

In conclusion, this study provides evidence of significant associations between ocular HOAs and axial eye growth, when controlling for confounding variables. Increased levels of total HO RMS, SA RMS and more positive primary SA $({{\rm{Z}}}_{4}^{0})$ and vertical trefoil $({{\rm{Z}}}_{3}^{-3})$ and negative oblique trefoil $({{\rm{Z}}}_{3}^{3})$ were associated with slower axial eye growth in children. These findings support the hypothesis that HOAs could provide a cue to eye growth in a vision dependent mechanism underlying refractive error development.

## Methods

### Subjects and procedures

This retrospective study analysed the data from 138 control participants (primarily myopic children wearing single-vision spectacles) who previously completed two-year longitudinal clinical trials (ROMIO^67^: 41; TO-SEE^68^: 23; HM-PRO^69^: 16; PR (a subset of Lee & Cho)^74^: 58) (Table 3). All studies were conducted in accordance with the tenets of the Declaration of Helsinki and approved by the Departmental Research Committee of the School of Optometry of The Hong Kong Polytechnic University with written informed consent obtained from both subjects and their parents before participating in the studies. The studies were also registered at ClinicalTrials.gov (ROMIO: NCT00962208; TO-SEE: NCT00978692; HM-PRO: NCT00977236; PR: NCT00978679).

**Table 3:** Description and baseline demographics (mean ± SD) of analysed subjects (n = 137) from ROMIO^67^, TO-SEE^68^, HM-PRO^69^ and PR (a subset of Lee & Cho study)^74^ studies.

	ROMIO	TO-SEE	HM-PRO	PR
Description	Low to moderate myopes	High astigmats	High myopes	Low hyperopes and myopes
Subject number	40	23	16	58
Age, y	9.2 ± 1.1	9.4 ± 1.6	10.5 ± 1.1	7.8 ± 0.8
Myopia, D	−2.23 ± 0.85	−1.97 ± 1.26	−6.34 ± 0.76	−0.01 ± 1.41
Astigmatism, D	−0.27 ± 0.34	−1.76 ± 0.61	−0.98 ± 0.35	−0.34 ± 0.38
SER, D	−2.36 ± 0.87	−2.85 ± 1.27	−6.84 ± 0.85	−0.18 ± 1.37
Axial length, mm	24.40 ± 0.85	24.19 ± 1.02	25.97 ± 0.53	23.32 ± 1.02

SER: spherical equivalent refraction.

Myopic subjects (sphere ≤ −0.50 D) were fully corrected with single-vision spectacles while a small proportion of hyperopic subjects (n = 24) were habitually uncorrected. All subjects underwent annual cycloplegic examination and data collection, at least 30 minutes after topical instillation of one drop of 0.5% proparacaine, 1% tropicamide and 1% cyclopentolate, each administrated 5 minutes apart. The effectiveness of cycloplegia was confirmed when there was no pupillary reflex and the accommodation amplitude was less than 2D. Measurements of axial length, HOAs and cycloplegic subjective refraction were collected by a masked examiner, to the treatment received by the subjects.

The axial length of each eye was determined as the average of five readings with a maximum difference of 0.02 mm and signal-to-noise ratio above 3.5, measured by a non-contact optical biometry based on the principle of partial coherence laser interferometer (IOL Master 500; Zeiss Humphrey Systems, Dublin, CA, USA). This device has good repeatability of axial length measurement in cyclopleged subjects of similar age and refractive error to those in our study^75^. Monochromatic ocular aberrations, calculated for a wavelength of 555 µm, were captured using a Hartmann-Shack aberrometer (Complete Ophthalmic Analysis System [COAS]; Wavefront Sciences Ltd., New Mexico, USA). During COAS measurements, room illumination was kept at minimum to avoid the influence of stray light. At least five wavefront measurements were taken for each eye and later averaged. Cycloplegic subjective refraction was also performed aiming for maximum plus/minimum minus for maximum visual acuity.

### Wavefront analysis

Wavefront data obtained from the COAS were fitted with a 6th order Zernike polynomial expansion. Using customized software, the averages of the coefficients of the Zernike polynomials were calculated, after rescaling to a set pupil diameter of 6 mm^76^. Refractive power maps^77^ were also generated to illustrate the refractive power distribution across the pupil based on the ocular HOAs (3rd to 6th order inclusive).

### Statistical analysis

All statistical analyses were performed using SPSS 23.0 software (IBM Corp., Armonk, NY, USA). Due to the presence of optical enantiomorphism (mirror symmetry) between the two eyes^78^ and the high degree of correlation between the two eyes for HOAs^79^, only the data from right eyes were included for analysis^35^. Since a natural logarithm of age provided a better fit when modelling the association between age and axial length, similar to previous studies of eye growth during childhood^54^, a logarithmic transformation of age was applied prior to adding other predictor variables. A LMM approach was then applied to first examine the influence of HO RMS on longitudinal changes in axial length and their associations with predictor variables over time with restricted maximum likelihood estimation. This analysis examined the effect of age upon axial length assuming a first-order autoregressive covariance structure. Individual subject’s slopes and intercepts were included as random effects as an unstructured covariance type in the model. Categorical predictor variables (sex) were included in the model as fixed factors and continuous predictor variables (time, spherical equivalent refraction [SER], HO RMS) were treated as covariates. The interactions between each different predictor variable with time were also included in the LMM to provide information about each factor and their associations with axial eye growth.

To provide further insights into the association between components of total ocular HOAs and axial elongation, two additional LMMs were performed to investigate if specific RMS metrics (SA RMS and coma RMS) or individual Zernike coefficients (3rd-6th orders) were significantly associated with axial length and axial eye growth, including categorical predictor variables (sex) as fixed factors and continuous predictor variables (time and axial length) as covariates. All LMMs were determined using a backward stepwise approach with the least significant factor removed with each iteration and the Akaike information criterion as the metric to compare the relative quality of each model^80^. Owing to the differences reported in HOAs between hyperopic and myopic eyes^39^, the above LMMs were also repeated excluding hyperopes (24 subjects with sphere > −0.50 D), but these yielded similar results. Therefore, the results presented are from analyses including all participants unless otherwise specified. A *P* value < 0.05 was considered as significant.
